# Contraceptive Behavior in Appalachia: Exploring Use, Nonuse, and Contraceptive Attitudes

**DOI:** 10.3390/ijerph20196862

**Published:** 2023-09-29

**Authors:** Samantha Auerbach, Kafuli Agbemenu, Rebecca Lorenz, Amy Hequembourg, Gretchen E. Ely

**Affiliations:** 1 School of Nursing, University at Buffalo, Buffalo, NY 14214, USA; agbemenu@buffalo.edu (K.A.); rlorenz@buffalo.edu (R.L.); ahequemb@buffalo.edu (A.H.); 2 College of Social Work, University of Tennessee, Knoxville, TN 37996, USA; geely@utk.edu

**Keywords:** contraception behavior, Appalachian region, acceptability, attitudes

## Abstract

Very little is known about contraceptive behavior in Appalachia, a large geographic region in the eastern United States where even basic prevalence estimates of contraceptive use/nonuse are lacking. This study characterizes contraceptive behavior among Appalachians, including contraceptive use, reasons for use, and methods used; contraceptive nonuse and reasons for nonuse; and attitudes about contraception, including acceptability. This is a secondary analysis of a subsample of survey data collected on sexual and reproductive health attitudes, behaviors, and needs among reproductive-age women (18–49 years) living in the Appalachian region (*n* = 332). Results identify rates of contraceptive use (66.6%) and nonuse (33.1%) among Appalachian residents. Methods used most frequently included those that did not require prescription (i.e., external condoms and natural family planning methods) though many reported the use of intrauterine devices (IUDs). Among nonusers, fear of side effects from contraception and ambivalence towards pregnancy were most commonly selected as the most important reason for not using contraception. Contraception was considered acceptable by this sample overall, and these acceptability attitudes were significantly associated with contraceptive behavior.

## 1. Introduction

Contraceptive behavior is complex. The decision to use or not use contraception is highly personal and reflective of a confluence of attitudes towards pregnancy, belief systems, personal abilities, and future goals, all of which are nestled within a social and structural context with varying degrees of contraceptive stigma and barriers to access. How people decide to use or not to use contraception may then be influenced by the particular sociostructural context in which they live. For example, contraceptive decision making may be particularly fraught in areas with restrictive state policy and strong sociocultural stigma [1]. Understanding contraceptive behavior and attitudes towards contraception is crucial for planning resource allocation and efforts to support and expand contraceptive access, and this information may then be especially important among those living in places where contraception is most restricted.

The Appalachian region of the United States, spanning portions of thirteen states, stretching from southern New York to northern Alabama, is a large geographic area offering a range of diverse experiences, though through the exploitation of the land and strategic under-resourcing, many areas within Appalachia are economically marginalized [2]. Residents are thought to share features of a culture or identity, especially in relation to rurality, ties to family and religion, connections to the land, and a sense of justice [2]. There are distinct inequities in reproductive health and heath access in Appalachia [3], in part due to structural barriers to accessing reproductive health services in the region. For example, many of the states containing Appalachian counties have passed hostile policies towards reproductive health services, such as greatly restricting abortion and dismantling the family planning safety net. Even prior to the *Dobbs vs. Jackson* ruling and subsequent outright bans on abortion, 11 of the 13 states containing Appalachian counties had so many restrictive laws for abortion that they were rated as having abortion-hostile policy [4]. Importantly, evidence suggests this hostile policy translated into residents’ perceptions that abortion is unattainable for them [5]. Policies that eliminate or limit family planning services, such as those that prohibit funds for entities that provide referrals for abortion services, have further worsened the lack of available contraceptive services in Appalachia [6]. Over 1.5 million Appalachian residents live in ‘contraceptive deserts,’ counties that lack adequate access to contraception due to a lack of providers or lack of availability of contraceptive methods [7]. Research from focus groups with community constituents, including Appalachian health professionals and service workers who live and work in the region, reported rural geography, the lack of transportation infrastructure, and the lack of providers as structural barriers to obtaining contraceptive care in the region [1].

Sociocultural barriers to contraceptive use in Appalachia may also impede reproductive health and access to services. For example, evidence suggests contraceptive stigma and the lack of contraceptive knowledge also hinder contraceptive care in the region [1]. Other barriers to contraceptive care in Appalachia include provider bias and mistreatment during health care encounters, specifically health care encounters where the person sought contraception [8]. Evidence highlights how health care providers, influenced by social and cultural norms related to religious and patriarchal views, offered contraceptive services considered to be dehumanizing and misogynistic, and consequently, many in the study did not want to return for care [8]. Additional evidence suggests people seeking contraception in Appalachia experience coercion related to their care. Swan et al. [9] found that more than one-third of Appalachian women reported health care provider coercion related to their contraceptive method in a recent study, including both pressure to use and not use contraception.

While the literature provides information on the various kinds of barriers to seeking reproductive health services in general and contraception specifically, very little attention has been given to contraceptive behavior or decision making in the Appalachian region. For example, no known studies have measured the rates of contraceptive use or nonuse among the general population of the Appalachian region. Two identified studies [10,11] were sampled from specialized populations, whose behavior may or may not be reflective of the general Appalachian population. For example, in these studies, contraceptive behavior was measured among incarcerated individuals in rural Appalachian jails (*N* = 193) [11], and Ely et al.’s study sample was comprised solely of self-identified ‘drug users’ (*N* = 400) [10]. Ely et al. [10] reported that nearly 69% of their sample did not use contraceptive methods in the six months prior to incarceration (*N* = 400), while Wenzel et al. [11] indicated that 60.2% of their sample reported using contraception ‘at least once’ in the three months prior to incarceration (*N* = 88). Chakraborty et al. [12] reported the preferred contraceptive method used in an Appalachian portion of Ohio, though information about contraceptive use and nonuse was not included in the main analysis.

Outside of Appalachia, contraceptive behavior is well characterized, with a large evidence base documenting contraceptive use prevalence rates among various populations and associations between sociodemographic factors and contraceptive use and nonuse, including race/ethnicity, educational attainment, and socioeconomic status [13,14,15]. However, contraceptive decision making remains poorly understood. Some evidence suggests decisions to use or not use contraception are associated with attitudes towards pregnancy, such as conflicting positive and negative feelings towards pregnancy, i.e., ambivalence [16,17], or perceptions of low control over becoming pregnant [18]. Other evidence shows attitudes towards contraception, such as contraceptive self-knowledge [19,20,21] and social acceptability/unacceptability [22,23], to be associated with contraceptive decision making. These attitudes have not yet been explored among Appalachian residents, however, where even basic prevalence rates of contraceptive behavior remain unknown.

In the context of this gap in knowledge of contraceptive behavior in this region, the current study aims to characterize contraceptive behavior among Appalachians, including contraceptive use, reasons for use, and methods used; contraceptive nonuse and reasons for nonuse; and acceptability attitudes about contraception. To our knowledge, this is the first contraceptive study to draw respondents from each of the five Appalachian subregions [24]. Characterizing contraceptive behavior in the Appalachian region is critical for identifying important factors in contraceptive decision making that can be leveraged into person-centered contraceptive counseling interventions tailored to the region, as well as provide a baseline measurement of this important health behavior upon which future work can build.

## 2. Material and Methods

### 2.1. Sampling and Recruitment

The current study is a secondary analysis of data collected as a broad survey of sexual and reproductive health attitudes, behaviors, and needs among reproductive-age women (18–49 years) living in the Appalachian region (*N* = 628). Using convenience sampling strategies of Facebook advertising targeted to Appalachian zip codes and groups, a purposive sample of women were recruited for an anonymous online survey. This method of recruitment was chosen for its strengths in targeted research on sensitive topics, and for its proven success in recruiting difficult-to-access Appalachian residents [25]. Interested participants followed a link from Facebook to a secure survey platform, REDCap [26], to complete the survey online. Participants who provided an email upon survey completion were compensated with a USD 10 retail gift card. Inclusion criteria included age 18–49 years, assigned female at birth, and living in an Appalachian zip code. For the purposes of this secondary analysis, participants were excluded if they were not currently at risk for unintended pregnancy. Thus, exclusion criteria included those: currently seeking pregnancy, in the immediate postpartum period (12 weeks), with diagnosed infertility, with a partner with whom pregnancy is not possible due to sex assigned at birth or use of sterilization methods, and not sexually active within the past 3 months [27].

All participants were anonymous. This research protocol was approved by the Institutional Review Board at the University at Buffalo.

Advertisements for the survey were active in November 2019 and ended when target enrollment was reached. Responses were checked for signs of fraudulent activity using recommended fraud detection techniques on survey meta-data, such as analysis of completion times and response patterns [28,29,30], and responses determined to be duplicate or fraudulent were removed from the study.

### 2.2. Measures

Contraceptive use was assessed with “are you currently using a method of birth control?” and with “yes/no” response options. Follow-up questions were presented using branching logic based on participant’s response concerning their current contraceptive use. Reasons for contraceptive use/nonuse were elicited by asking participants to “choose the ONE most important reason for you using/not using birth control.” Participants were presented with eleven fixed-response options (e.g., “how effective it is at preventing pregnancy”, “because my partner doesn’t want to use a birth control method”).

A composite score measuring contraceptive acceptability was constructed from 7 items assessing perceptions of contraceptive acceptability (Cronbach’s alpha = 0.79). Sample items included: “Abstinence is the only acceptable form of birth control” and “Birth control is bad/immoral,” with possible responses ranging from 1 (Strongly agree) to 5 (Strongly disagree). Higher scores indicated greater contraceptive acceptability. This measure is currently undergoing formal psychometric evaluation [31]. Attitudes toward current contraceptive method were assessed using responses indicating level of agreement with the statement, “I am happy with my current birth control method”.

Demographic variables were selected based on their relevance to contraceptive behaviors, as identified in prior studies [13]. Annual household income was assessed through a series of categorical response options (USD 0–14,999; USD 15,000–29,000) and scored to reflect low (USD 0–49,000) and high annual household income (USD 50,000+). Religious identity was assessed by responses to “I consider myself a member of a religious group”, where ‘strongly agree’ and ‘agree’ responses were categorized as ‘religious’ and ‘strongly disagree’ and ‘disagree’ responses were categorized as ‘non-religious’. Rural residence was defined by Beale rural–urban continuum codes ranging from four to nine [32]. All other Beale codes were scored to represent non-rural residence. Appalachian subregion was defined by the Appalachian Regional Commission [24].

### 2.3. Analysis

Prevalence of contraceptive use/nonuse, associated reasons for use/nonuse, and attitudes towards contraception were assessed using frequency analysis. Relationships between contraceptive behavior and sociodemographic factors or Appalachian subregion were assessed using chi-square tests of association. Relationships between contraceptive acceptability and sociodemographic characteristics, current contraceptive use, and Appalachian subregion were evaluated using *t*-tests for continuous variables, chi-square tests for dichotomous variables, or one-way ANOVA for categorical variables. All analysis was completed in SPSSv.28 (IBM Corp., Armonk, NY, USA) [33].

## 3. Results

### 3.1. Sample Description

The sample was reduced to *n* = 332 after applying the inclusion/exclusion criteria. The mean age of the participants was 32.5 years (*SD* = 6.3; range: 20–49 years). The majority of the participants identified as white (87.8%), with the remaining participants identifying as American Indian/Native American (5.1%), Black/African American (4.2%), Latina/o/x (1.8%), and Asian/Pacific Islander (0.9%). The sample was nearly evenly split between rural (49.1%) and non-rural (50.9%) residences. Half of the participants (51.7%) reported a household annual income of USD 49,000 or less, and nearly all (97.0%) reported annual income of USD 100,000 or less. Nearly ninety percent of participants (89.3%) reported current health insurance coverage. Just over three-quarters of respondents (76.8%) reported being married or partnered, and over half (54.3%) identified as religious.

### 3.2. Characterization of Contraceptive Use

Contraceptive use prevalence and reasons for use can be found in Table 1. The most commonly reported methods of contraception used were: external condoms (25.4%); intrauterine devices (IUD, 16.7%); natural family planning methods (rhythm method, calendar method; 13.7%); and oral contraceptive pills (12.5%).

### 3.3. Characterization of Contraceptive Nonuse

Contraceptive nonuse prevalence and reasons for nonuse can be found in Table 1. The most commonly reported reasons for nonuse were worry about the side effects (25.5%) and ambivalence to becoming pregnant (24.5%).

### 3.4. Sociodemographics and Contraceptive Use/Nonuse

There was a statistically significant association between respondent age and contraceptive use/nonuse, with older participants less likely to currently use a contraceptive method than younger participants (*t*(187) = 2.519, *p* = 0.006, *d* = 0.31). Furthermore, participants with lower incomes (χ2(5) = 24.513, *p* < 0.001) and those without health insurance (χ^2^(1) = 11.456, *p* < 0.001) were significantly less likely to currently use a contraceptive method. No associations were observed between contraceptive use or nonuse and religious identity, racial identity, relationship status, rural residence, or Appalachian subregion.

### 3.5. Contraceptive Acceptability and Contraceptive Use/Nonuse

Among current contraceptive users, nearly half (47.7%) reported being happy with their current method (47.7%). Among both users and nonusers, nearly one-third (29.6%) reported wanting to use a different method than their current one or wanting to begin a method if they were not currently using one. Associations between contraceptive use and nonuse and acceptability scores are described in Table 2.

### 3.6. Contraceptive Acceptability and Sociodemographics

Contraceptive acceptability scores were significantly different across Appalachian subregions, *F*(4,623) = 6.225, *p* < *0*.001. Post-hoc analyses using Tukey HSD indicated that the mean acceptability scores were significantly lower among residents of South–Central region (28.0) compared to the North–Central (29.9) and Central (29.9) regions. Additional associations between acceptability scores and sociodemographic characteristics are described in Table 2.

## 4. Discussion

This study is one of the first to characterize contraceptive behavior among Appalachian residents, as well as describe reasons for contraceptive use, nonuse, and attitudes towards contraception. We found that two-thirds of our sample utilized contraceptive methods at the time of the survey. The proportions of our sample using or not using contraceptive methods are similar to the overall rates of contraceptive use (65.3%) and nonuse (34.7%) among women in the United States, which was surprising given the formidable barriers to contraceptive care present in Appalachia [34]. Our sample’s rates of contraceptive use and nonuse are also similar to the rates reported in one study of Appalachian residents (60.2% and 39.8%, respectively) in the three months prior to incarceration [11], but nearly reverse the use and nonuse rates (31.3% and 68.7%, respectively) in the other available study of Appalachian residents prior to incarceration [10].

Of contraceptive users, more than one-third reported using either external condoms (25.4%) or natural family planning methods (13.7%), such as the rhythm method or calendar method, while smaller proportions of users reported using oral contraceptive pills or other methods. The prevalent use of these less effective contraceptive methods is incongruous with the fact that more than one-quarter of the sample reported the single most important factor when choosing their contraceptive method was efficacy at preventing pregnancy. The use of more easily accessible methods (i.e., available without a prescription or visit to a health care provider) may reflect the relative lack of contraceptive services in the area, as rates of external condom and natural family planning method use among a national sample are notably lower [7,35]. Interestingly, however, rates of IUD use were high (16.7%), which indicates an important proportion of residents may be accepting of long-acting reversible contraceptive (LARC) methods that require health care provider insertion.

These potential preferences among residents of Appalachia has important resource allocation implications. For example, enhancing access to condoms, evidence-based information regarding natural family planning methods, and assessing interest in non-prescription contraceptive methods are all possible strategies for tailoring contraceptive counseling for residents of this region. Attitudes towards having oral contraceptive pills available without prescription may be particularly important in Appalachia given the use of non-prescription methods, and this may be a priority policy area in the future. It is critical, however, to assess interest in other methods and barriers to obtaining other methods, to ensure users of external condoms and natural family planning methods are not utilizing those methods simply due to lack of access to other alternatives.

Additionally, the prevalent use of IUDs as a contraceptive method in this sample may be considered in the context of high rates of reported mistreatment and contraceptive coercion during health care encounters in Appalachia [8]. Coercion to use LARC methods from health care providers have been established, including perceptions that providers disproportionately recommend LARCs to those most socially marginalized [36]. These perceptions of the coercive use of LARC have also been reported among residents of Appalachia, particularly in relation to those using opioids [1]. Further work to clarify contraceptive decision-making processes among this population, particularly related to LARC methods, is needed to more fully understand this finding, given the evidence of contraceptive coercion experienced in the region.

Among contraceptive nonusers, fear of the side effects from contraceptive methods and ambivalence towards pregnancy were most commonly selected as the most important reason for not using contraception. These attitudes towards pregnancy and contraception have been associated with contraceptive behavior in a variety of populations in the research literature, but to our knowledge, this is the first account from an Appalachian sample. These results are a critical first step toward the identification and development of regionally-responsive contraceptive counseling efforts. For example, results suggest that effective regional educational campaigns could highlight expected contraceptive side effects versus common myths and misperceptions about side effects to provide opportunities for increasing self-knowledge of contraception. Health care and social service providers engaging in contraceptive counseling should consider discussing pregnancy ambivalence in the content of contraception, when appropriate. Moreover, results suggesting those with low income and those lacking health insurance are significantly more likely to not use contraception highlight the need for regional policy goals to include the state expansion of Medicaid eligibility and enforcement of the violation of the Affordable Care Act’s contraceptive coverage requirement to ensure access to contraceptive services [37,38].

Contraception was considered acceptable by this sample overall, despite the restricted and stigmatized nature of contraceptive care in the region. Contraceptive acceptability scores were also significantly associated with contraceptive behavior, whereby attitudes that contraception is acceptable were significantly more likely among those who were currently using contraception. While this association is not necessarily surprising, we feel this indicates the potential utility of this scale to capture and measure attitudes towards contraception that are integral to behavior, and thus should be more robustly evaluated in the future. These contraceptive acceptability attitudes were also significantly less likely among those who self-identified as religious, those residing in rural locations, and those living in the South-Central Appalachian subregion (counties in eastern North Carolina, western Tennessee, and western Virginia), providing important information for tailoring contraceptive education and counseling when addressing contraceptive behavior in these groups. For example, understanding the increased likelihood of someone perceiving contraception as unacceptable can direct contraceptive counseling techniques to honor and explore these acceptability attitudes and their relation to behavior, thereby better centering Appalachian residents’ needs.

Unlike contraceptive acceptability attitudes, contraceptive behavior was not associated with religious self-identification or rural residence in this sample, reflecting evidence from nationally-representative samples reporting contraceptive behavior was not associated with religiosity [39]. The fact that acceptability attitudes were related to sociostructural factors of religiosity and rurality, but contraceptive behavior was not, suggests that the contexts of religiosity and rural residence may influence attitudes about contraception, but that these attitudes alone do not translate into contraceptive use or nonuse behaviors. Moreover, this result implies that decisions to use or not use contraception may occur in direct opposition to attitudes about contraception among some subsets of the sample, indicating a cognitive inconsistency that can be further explored in future qualitative work [40]. More research is needed to understand the complex relationship between contraceptive attitudes and contraceptive behavior among those who identify as religious or live in rural locations.

While this study provides many novel insights about contraceptive behavior among residents of Appalachia, several limitations should be noted. The cross-sectional design of the study and the non-probability sampling methods limit our ability to draw causation and generalize these results to the larger Appalachian population. The homogenous racial identities reported by this sample also limits the generalizability of results, as they do not reflect the racial composition of the larger Appalachian region [41]. Similarly, this sample had a younger mean age, lower mean household income, and greater proportion of living in rural areas compared to the Appalachian population as a whole [41]. The contraceptive acceptability scale is currently undergoing formal psychometric validation [31], however, it had not yet been validated at the time of this analysis. This work was completed prior to the *Dobbs vs. Jackson* ruling, and thus reflects a snapshot of contraceptive behavior and attitudes in Appalachian residents from a time when abortion access remained federally protected, though already significantly difficult in most of the states from which this sample was derived.

## 5. Conclusions

In this first-of-its-kind characterization of contraceptive behavior and attitudes among residents of Appalachia, contraception was used by the majority of the sample; however, it tended to favor less effective, but more easily accessible methods. Given the presence of social and structural barriers to contraceptive care in Appalachia [7], the similar prevalence of contraceptive use in our sample versus national samples suggests adaptation and contraceptive resourcefulness among residents of Appalachia that warrants further exploration. The prevalent use of methods that do not require prescription is perhaps related to these barriers and the presence of contraceptive deserts in the region, but more work is needed to determine this, particularly given the high rates of IUD use. Contraceptive use was considered acceptable, overall, suggesting the many barriers to accessing care may not have impacted attitudes towards contraception in this sample. Acceptability attitudes were associated with contraceptive use/nonuse, whereby those with higher acceptability scores were more likely to use contraception, suggesting the need for the further development and validation of this scale. Taken together, these results serve an important role in the literature base of contraceptive behavior in Appalachia and can be used to tailor contraceptive counseling, inform educational messaging, and guide contraceptive policy decisions in the region.

## Figures and Tables

**Table 1:** Contraceptive Use, Nonuse, and Reasons for Use/Nonuse Among Residents of Appalachia (*N* = 332).

	Contraceptive Users (%)		Contraceptive Nonusers (%)
Do you currently use a method of birth control?			
Yes	66.6	No	33.1
Which one factor is most important to your decision to use/not use birth control?			
	Use		Nonuse
Efficacy	42.5	Worry about side effects	25.5
Safety	21.7	Ambivalent to becoming pregnant	24.5
Ease of use	14.0	Do not expect to have sex	16.4
Potential side effects	7.7	Do not think you should try to control pregnancy	5.5
Long-term pregnancy prevention	5.4	Perceived subfertility	5.5
Cessation of menses	2.7	Perceived low risk of becoming pregnant	4.5
STI/HIV protection	2.3	Partner does not want to use method	1.8
Control of heavy bleeding	1.8	Unable to get method	0.9
Method(s) Used			
External condoms	25.4		
Intrauterine device	16.7		
Natural Family Planning (rhythm method, calendar method)	13.7		
Oral contraceptive pills	12.5		

**Table 2:** Contraceptive Acceptability Scores and Association with Sociodemographic Characteristics Among Residents of Appalachia (*N* = 332).

	Acceptability Scores	*p*	Cohen’s d
Mean (SD)	28.6 (4.5)		
Contraceptive Users	29.3 (4.6)	0.032 *	−0.17
Contraceptive Nonusers	28.5 (4.4)		
Rural	28.4 (4.7)	<0.001 ***	−0.28
Non-Rural	29.6 (4.1)		
Religious	28.0 (4.8)	<0.001 ***	−0.57
Not Religious	30.5 (3.9)		
Low Income	28.8 (4.3)	0.641	−0.04
High Income	29.0 (4.8)		

* *p* ≤ 0.05; *** *p* ≤ 0.001.

## Data Availability

The data presented in this study are available on request from the corresponding author. The data are not publicly available due to this being a secondary analysis of previously collected data.
